# Transoral Robotic Surgery for the Salvage of Primarily Irradiated Oropharyngeal Squamous Cell Carcinomas Recurring at the Base of the Tongue: A Small Monoinstitutional Series

**DOI:** 10.3390/jpm15090419

**Published:** 2025-09-03

**Authors:** Samuele Frasconi, Davide Rizzo, Roberto Gallus, Nikolaos Machouchas, Sergio Cannova, Dan Marian Fliss, Jacopo Galli, Francesco Bussu

**Affiliations:** 1 Otolaryngology Division, Sassari University Hospital, viale San Pietro 10, 07100 Sassari, Italy; drizzo@uniss.it (D.R.); nikolaos.machouchas@aouss.it (N.M.); fbussu@uniss.it (F.B.); 2 Department of Medicine, Surgery and Pharmacy, Università degli Studi di Sassari, 07100 Sassari, Italy; s.cannova@studenti.uniss.it; 3 Division of Otolaryngology, Mater Olbia Hospital, 07026 Olbia, Italy; roberto.gallus@materolbia.com; 4 Division of Otolaryngology, Head & Neck and Maxillofacial Surgery, Tel-Aviv Sourasky Medical Center, Tel-Aviv 6423906, Israel; iritst@tlvmc.gov.it; 5 Institute of Otorhinolaryngology, Policlinico Agostino Gemelli, Università Cattolica del Sacro Cuore, 00168 Rome, Italy; jacopo.galli@unicatt.it

**Keywords:** oropharynx, base of tongue, robotic surgery, treatment deintensification, salvage surgery, post radiotherapy recurrences, TORS

## Abstract

**Background/Objectives**: Recurrences of squamous cell carcinoma (SCC) at the base of the tongue (BoT) after primary radiochemotherapy (RT-CHT) are associated with low survival rates, poor functional outcomes, and high morbidity following salvage surgery. Transoral robotic surgery (TORS) has emerged as a less invasive alternative to open surgical approaches. This study aims to describe our clinical experience with TORS in patients with BoT SCC recurrence after RT-CHT, focusing on oncological outcomes—relapse-free survival (RFS) and disease-specific survival (DSS)—as well as functional outcomes, particularly swallowing function. **Methods**: We conducted a retrospective review of four patients who underwent salvage TORS for BoT recurrence between September 2013 and September 2014 at a single tertiary referral center. All patients had been previously treated with primary RT-CHT for oropharyngeal squamous cell carcinomas. Oncological events (recurrence, death) and functional endpoints (dietary limitations, MD Anderson Dysphagia Inventory [MDADI] scores) were retrieved from medical records. **Results**: Four patients were included. All achieved unrestricted oral intake by one month post-TORS, showing functional improvement compared to their preoperative status. Three of the four patients remained free of locoregional recurrence during follow-up. No major perioperative complications were reported. **Conclusions**: In selected patients with BoT SCC recurrence after primary RT-CHT, TORS may offer a viable and less morbid salvage treatment option with favorable early functional outcomes and acceptable oncologic control. Based on both our institutional experience and the supporting literature, we propose selection criteria to guide TORS indication in this clinical setting.

## 1. Introduction

It has been estimated that nearly 60,000 individuals (specifically 59,660) will be diagnosed with head and neck cancer (HNC) in the United States in 2025, with the vast majority of cases representing squamous cell carcinoma (HNSCC). This burden will translate into approximately 12,770 cancer-related deaths over the same period [1]. Over the last twenty years, the incidence of HNC has shown a sustained upward trend, and projections suggest that if current trajectories persist, the number of new cases could potentially double by 2030 [2]. Presently, HNSCC constitutes approximately 3.5% of all malignancies diagnosed in the U.S. population, and about 4% of cancers across Europe [3].

Traditionally, the most recognized etiological factors for HNSCC have been tobacco use and chronic alcohol consumption [4,5]. Nonetheless, the recent and pronounced rise in HNSCC incidence observed in many Western countries can largely be attributed to oncogenic strains of human papillomavirus (HPV), the so-called high-risk (HR) genotypes [6,7,8,9,10] which cause oropharyngeal squamous cell carcinoma (OPSCC) [11,12]. The rate of HPV-related OPSCC has exceeded that of HPV-unrelated cases since 2004 in the US and in some European areas [10,13]. Therefore, as HR HPV infection in OPSCC is notably associated with markedly better survival rates [14,15,16], deriving at least in part from a higher sensitivity to proapoptotic agents (i.e., irradiation and chemotherapy) [17,18], it is easy to postulate that so-called “HPV epidemics” contributed to the sharp improvement of the OPSCC prognosis in recent decades [18,19]. The assessment of HPV-driven carcinogenesis has been included in the work-up of OPSCC [14,20,21] and has been the trigger for an intense ongoing debate concerning treatment deintensification in HPV-related OPSCC [22,23,24].

In fact, the recognition of HR HPV infection as a strong predictive and prognostic factor in OPSCC has led to its incorporation into the TNM classification, effectively distinguishing HPV-positive tumors from HPV-negative tumors as biologically and clinically distinct entities. This distinction has, in turn, driven the growing interest in treatment deintensification strategies.

Among the options for treatment deintensification, transoral surgery, in particular using surgical robots (TORS: transoral robotic surgery), is currently increasing in popularity; in selected HPV-positive patients [25], treatment deintensification is currently the most popular indication for TORS in OPSCC.

For this reason, most of the cases in the recent series describing the advantages of TORS in OPSCCs are HPV+ primary cases [26,27,28]. These studies report better survival rates and functional results in TORS patients than in patients treated with non-surgical modalities, thus confirming the advantages of a less invasive approach, a clearer and wider 3D view than transoral laser microsurgery, the use of miniaturized tools with tremor filtration, and the management of the “blind corners” of the pharynx in a frontal view using a 30° endoscope and the articulated arms [29].

However, treatment deintensification through TORS on primary OPSCC should still be validated in ongoing clinical trials and compared with other strategies [23].

On the other hand, even if the comparison between external approaches and TORS in the management of recurrent OPSCC has been less extensively evaluated, similar oncological outcomes, but markedly lower complication rates and long-term morbidity in patients treated through TORS have been reported [30].

The aim of the present study was to verify these last figures in our preliminary clinical experience concerning recurrent squamous cell carcinomas at the base of the tongue (BoT), and to outline, using the available literature data, specific, rational and oncologically safe indications for TORS in such a salvage setting. The homogeneity of the series (all cases were post-irradiation recurrences at the base of tongue) allowed us to reduce biases deriving from different patterns of spread/surgical techniques as well as adjuvant treatments.

## 2. Materials and Methods

### 2.1. Patient Characteristics

All the patients previously treated for OPSCC using primary radiochemotherapy at Policlinico Gemelli, Università Cattolica del Sacro Cuore, and diagnosed with recurrence at the BoT between September 2013 and September 2014, who underwent salvage TORS were included. Informed consent was obtained from all subjects involved in the study.

All the head and neck cancer patients were evaluated by a multidisciplinary head and neck tumor board, comprising otolaryngologists, radiation oncologists, medical oncologists, radiologists, nuclear medicine specialists, and plastic surgeons, which managed every clinical recommendation through the thorough evaluation of acquired clinical and imaging data.

### 2.2. Diagnostic Work-Up

All the patients underwent early diagnosis of the recurrence in a multidisciplinary head and neck follow-up ambulatory of the institution. In all cases, the recurrence was first suspected at the physical examination by the otolaryngologists and was histologically confirmed after a complete imaging work-up, including a PET-CT and an MRI. The therapeutic recommendation, based on the criteria described in Table 1, and the surgical planning were achieved through the joint evaluation of the imaging by the surgeon and the radiologist. In comparison with the previous criteria proposed by White et al. [30] back in 2013, we specifically assessed anterior spread (no more than 2 cm beyond the lingual V, and relied on the rT, possibly resorting to histological mapping to reliably assess the extent of the recurrence and therefore plan the surgical resection well in advance.

### 2.3. Surgery

**Figure 1:**
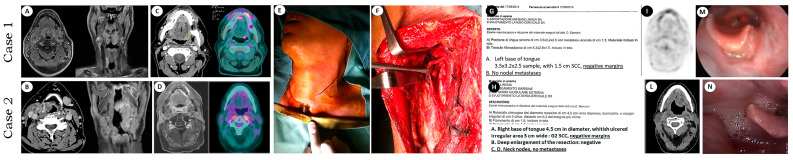
Case 1 was primarily affected with an HPV-positive (detected by mRNA on fresh sample) SCC at the base of the tongue (with a prevalent extension on the left side) (**A**), and the patient had not ever smoked or drunk. On the contrary, Case 2 presented with two synchronous HPV-negative SCCs at the left base of the tongue and the right tonsil (**B**), and he was a heavy smoker and drinker (40 pack/year) who has not quit even after the salvage TORS. The recurrence at the base of the tongue in both Cases 1 and 2 was first suspected on palpation. Case 1 was clearly confirmed through PET CT and morphological imaging (**C**), followed by biopsy. In Case 2, both PET CT and MRI were negative (**D**) and a definite diagnosis of recurrence was obtained only through histopathology; multiple biopsies were performed in oropharyngeal landmarks to precisely define the extent of the salvage resection and to exclude the recurrence of the second tonsillar primary. In both cases, the robotic procedure was performed following subisthmic tracheotomy (**E**), neck dissection, and lingual artery ligature (**F**). The resection margins were clear in both cases (**G**,**H**). Notably, the histopathology report demonstrated recurrence with a greater major diameter in Case 2 than in Case 1 (**G**,**H**). Both patients swallowed and spoke normally and experienced no recurrence of the OPSCC. Case 1 is currently alive after 9 years, while case 2 died 6 years after for a massive stroke ((**I**,**L**) postoperative imaging, (**M**,**N**) postoperative endoscopic view; see also Table 1).

The transoral robotic surgery (TORS) procedure employed in our series generally followed the approach described by Moore et al. [31], with the preliminary steps including a sub-isthmic tracheostomy and cervical lymph node dissection. In cases classified as rN0, a monolateral selective neck dissection was performed, targeting levels IIb to Va, whereas in the rN2c patient, a comprehensive bilateral dissection was conducted. During the neck phase, the lingual artery ipsilateral to the oropharyngeal tumor was identified and ligated to reduce bleeding risks (Figure 1).

General anesthesia was managed with a neuromuscular blockade specifically induced to facilitate transoral exposure. Initial setup involved dental protection and the insertion of a mouth gag to adequately expose the oropharyngeal lesion. Once exposure was confirmed, the Da Vinci Si Surgical System (Intuitive Surgical, Inc., Sunnyvale, CA, USA) was deployed on the patient’s left side. A 30° dual-channel endoscope was inserted to provide a panoramic view of the tumor field. The left robotic arm was fitted with a 5 mm EndoWrist Schertel grasper (Intuitive Surgical, Inc.), while the right robotic arm carried a 5 mm monopolar spatula cautery instrument. Both robotic arms were advanced carefully under direct vision.

The retractor blade was gently positioned in an anterior direction to ensure optimal visualization of the tumor’s anterior margin and to expose the inferior extent of the neoplasm as fully as possible. Mucosal incisions were initiated at the anterior border of the lesion to facilitate posterior displacement into the endoscopic field. An additional incision was placed at the paramedian line to permit the resection of part of the contralateral BoT, allowing for broader oncologic margins. These steps also exposed the hyoid bone without provoking significant hemorrhage.

Once anterior and medial incisions were completed, the robotic retractor was employed to grasp healthy tissue at the anterior edge of the specimen, applying countertraction that greatly assisted in the dissection of the remaining tumor. The precise trajectory of the surgical cuts was determined by tumor extent. Resection proceeded through adjacent soft tissues that appeared to be normal upon inspection in order to achieve complete excision. The surgical field included the removal of the ipsilateral palatine tonsil. The hypoglossal nerve was always preserved.

Thanks to the enhanced maneuverability afforded by robotic instrumentation which is particularly advantageous for accessing the tongue base and for manipulating the tumor in various planes, and to the magnified 30° endoscopic visualization, the entire tumor could always be resected *en bloc*. Real-time intraoperative consultation with the pathologist via frozen section analysis increased the reliability of ensuring radical excision, though in all the present cases, frozen samples from the surgical bed were negative. Nonetheless, additional tissue margins adjacent to the tumor site were dissected and sent for histologic evaluation to confirm adequate clearance whenever visual inspection, physical palpation, or histopathological evidence suggested the possibility of residual disease.

### 2.4. Outcomes

All clinical and pathological staging parameters were extracted from medical records and histopathological reports. Oncological outcomes were assessed from the date of salvage surgery and included overall survival (OS), disease-specific survival (DSS), and relapse-free survival (RFS). Information on treatment-related toxicities—encompassing both short-term surgical complications and long-term sequelae—was also collected from patient records. Particular attention was given to functional outcomes, with data retrieved on swallowing function, the need for percutaneous endoscopic gastrostomy (PEG), and tracheostomy dependence. When available, patient-reported outcome measures (PROMs) were also analyzed, especially regarding dysphagia, using the MD Anderson Dysphagia Inventory.

### 2.5. Ethical Considerations

The current study was conducted in accordance with the ethical standards of the Declaration of Helsinki. According to the policies of our institutions, this case series did not require Institutional Review Board (IRB) oversight. This exemption is based on the fact that the patients were not subjected to any procedure with prior research intent, the series does not involve systematic investigation methods such as statistical analysis, and the report simply describes noteworthy clinical treatments, presentations, or outcomes. Additionally, no identifiable patient information has been included. Since the data were analyzed with a retrospective design, in this case, mandatory ethical approval is not required under Italian law (GU No 76; 31 March 2008).

## 3. Results

Four patients corresponded to the inclusion criteria and were included in the selected period. Patient and tumor characteristics, together with oncological and functional outcomes, are shown in Table 2. Median follow up has been 84 months, at 5-years OS = DSS = RFS = 75%.

All the surgeries resulted as R0 (negative margins) at the final histopathology report. For this reason, and considering the previous full dose of radiochemotherapy, further adjuvant treatment was never recommended by the tumor board in the present series.

None of the patients were lost to follow up, and none of the patients showed distant metastases. Three out of four cases obtained locoregional control. Two are currently alive with no evidence of the primary oropharyngeal disease, one of them is currently undergoing therapy with immune checkpoint inhibitors for a second primary tumor (lung adenocarcinoma), one died due to intercurrent cardiovascular disease 6 years after the procedure with no evidence of disease, and one case died of recurrent disease after a second transmandibular salvage. The HPV positivity rate in this small series was 25% (Case 1, Figure 1).

No significant postoperative complications nor long-term sequelae have been recorded.

The cuffed cannula was substituted in all patients with a cuffless cannula (with a cap) within the 4th postoperative day, and in all cases, the temporary tracheostomy underwent closure within the 10th postoperative day. All patients reported oral feeding without limitations compared with the pre-salvage status, at 1 month after the robotic resection. None of the survivors experienced drastic swallowing or breathing impairment requiring gastrostomy and/or a new tracheotomy.

## 4. Discussion

The main limitation of the present study is clearly the very small sample size. Other limitations that detract from the possibility to draw definitive conclusions which lead us to consider our results as preliminary include the absence of comparison group(s) and the retrospective design with relative potential selection bias.

However, the results of the present preliminary series of salvage TORS for recurrent OPSCC at the BoT are extremely encouraging and consistent with the literature [30].

The most popular indication to TORS in OPSCC is currently the primary HPV+ lesion, with the aim/justification of deintensification, which should still be validated in ongoing clinical trials and compared with other strategies [23].

In particular, some critical questions still need to be answered before really considering TORS as the standard of care for primary HPV + OPSCC:

- Is treatment deintensification through primary surgery rational for treating notoriously radiosensitive malignancies, such as HPV-related OPSCC? Is the survival obtained through TORS in this group really equivalent to that obtained with chemoradiation?
- In case of recurrence, is the pattern of spread of the recurrence and therefore the salvageability of patients previously treated through TORS similar to that of those who exclusively underwent radiation?
- How many OPSCC patients treated through TORS will require adjuvant treatment? Is the long-term toxicity from a single modality treatment, even at higher doses, really greater than that from a combined treatment?

In fact, the last question has been addressed by the Orator 1 randomized trial, with inconclusive findings, while the first one has been addressed by the Orator 2 randomized trial which has been interrupted for excessive acute morbidity (perioperative deaths) in the surgical arm [32].

Instead, a situation, as in the present series, in which transoral surgery, particularly TORS, would otherwise require a more invasive alternative, characterized by much greater morbidity, is the recurrence after primary radiochemotherapy [33]. Indeed, in these cases, the most validated option is a wide resection using a transmandibular approach with the need for immediate reconstructive procedures [20,34,35] and associated with high complication rates and highly relevant long-term morbidity and function impairments [30,36,37].

Our study confirms TORS as a valid alternative from an oncological point of view. However, in oncologic surgery involving the head and neck as well as other sites in humans, the precise recommendation and meticulous planning, relying on shared and evidence-based guidelines, are clearly the most decisive parameters for success. In the present study, we analyzed recurrences/persistence of SCCs at the BoT of individuals primarily treated using radiotherapy (with or without chemotherapy). This situation could occur in 25 to 50% of primarily irradiated OPSCC (strongly depending on the HPV positivity rate), which, as described above, is one of the most frequent head and neck malignancies and is certainly the condition with the clearest increase in incidence in head and neck oncology. Thus, as clinicians will often encounter such situations, the critical issue is to provide reliable criteria for choosing the proper salvage surgery, which, when feasible, is likely the only remaining possibility to save these patients. In some cases, TORS might be an oncologically safe option [30,38], enabling good early and long-term functional results and a short postoperative length of hospital stay [28,39,40].

Most TORS procedures described in the literature have been performed for primary cases [26,27]. Yet, even if the anatomy is the same as that observed in primary cases, the salvage of post-irradiation recurrences should be separately considered, as this condition presents a completely different situation due to the tissue modifications, and markedly higher complication rates that the surgeon will encounter, and most of all due to the attitude the tumor board must have in recommending the salvage treatment. In fact, OPSCCs are radiosensitive malignancies, and in TORS series, made up mostly of primarily operated cases, adjuvant radiochemotherapy is often performed, particularly because of positive/close margins [21,30], with a sure prognostic advantage [27,41]. On the other hand, effective adjuvant radiotherapy is usually not feasible in post-irradiation recurrences; therefore, the multidisciplinary evaluation and preoperatory planning should be even more carefully conducted and aimed especially at excluding positive margins, which in such cases are almost a death sentence and absolutely cannot be afforded.

The series described in the retrospective study of White et al. included both post-irradiation and post-surgical salvages in the whole oropharynx, without specifying the subsite. In contrast, the present small series includes only post-irradiation recurrences centered on the BoT, which are the most frequently amenable to TORS [30].

In general, these cases can be more frequently safely salvaged through TORS than recurrences in other subsites (particularly in the tonsil) for anatomical reasons (internal the and common carotid are farther, and it is often possible to obtain wider margins following the described planes of dissection), and for more predictable and relatively constant patterns of spread than in post-surgical relapses.

Therefore, the present small but homogeneous series, with relatively typical and clearly defined features, seems particularly suitable to discuss the selection criteria for salvage TORS. Some of these criteria are obvious and have practically been constantly adopted and described in the literature [30,42,43], such as the absence of bone involvement and trismus and a sufficient mouth opening (even if a consensus about clear objective parameters to predict the adequacy of the mouth opening for a TORS procedure has not yet been reached) (Table 1).

Other principles, such as the need to preserve at least one lingual artery in the procedure, are completely shareable, but this fact does not necessarily imply that resecting a BoT SCC crossing the midline is always contraindicated. Indeed, tumor proximity with both lingual arteries during surgery places the tongue at risk of devascularization especially in previously irradiated cases [44]. Therefore, a careful diagnostic work-up can identify the relationships between the mass and the arteries and assess the potential to preserve one of the vessels during TORS (Table 2). Functional issues, particularly concerning worse swallowing recovery, when more than half of the BoT is resected, which are of course fully justified under a theoretical point of view, are not confirmed in the present study, as swallowing recovery was fast and complete in this small series. Some parameters, particularly the anterior spread of the lesion, have not been considered in previous papers, even if they might cause clear limitations to TORS: the involvement of the hard palate and a more than 2 cm extension anterior to the lingual V have been our exclusion criteria (Table 1).

In providing our recommendation to salvage TORS, we ultimately relied on the diagnostic work-up and restaging of the recurrence, but not on the primary lesion. This issue has never been specifically addressed in previous studies, but in our opinion it is crucial; indeed, if we accept that the recommendation is based on the rT, we could use TORS to treat small recurrences/residuals from bulky primary lesions with a good but not complete response to radiotherapy, and in these patients, as in the present series, we could expect relatively high survival rates and good functional outcomes. The risk of further recurrences and failure due to subclinical multifocal residual disease can be decreased through seriate preoperative biopsies also in areas that are apparently free of disease after irradiation but are involved at primary clinical presentation. We believe that the role of frozen sections should be limited as much as possible, while “histological mapping” with definitive reports for the discussion at the tumor board should be included in the preoperatory work-up for use in therapeutic decisions and surgical planning.

In one of the cases examined in the present study (Table 2, Figure 1, Case 2), “histological mapping” was extensively used for therapeutic recommendation and surgical planning, in the face of substantially negative radiological and endoscopic findings, based upon the findings at physical exam/palpation. Therefore, we do not feel that the difficult visualization and/or palpation of margins are necessarily contraindicators of TORS, particularly when “histological mapping” demonstrates that the disease is confined to the BoT, which is resectable *en bloc* (Table 1). Also, this case supports the primacy of clinical evaluation and the surgeon’s judgment over any instrumental methodology/imaging technique.

The selection criteria proposed in this study (Table 1) provide a pragmatic framework for identifying post-irradiation BoT SCC recurrences that are amenable to TORS with curative intent. By integrating radiological, anatomical, and surgical considerations, they aim to maximize oncological safety while preserving function. In clinical practice, these criteria may support multidisciplinary teams in stratifying patients toward minimally invasive salvage options. When carefully applied, they may help reduce the morbidity that is traditionally associated with transmandibular approaches without compromising disease control, with incomparably better functional results and recovery and hospitalization times compared with more standardized options, such as transmandibular approaches, followed by reconstructive procedures (regional or free flaps).

## Figures and Tables

**Table 1:** Criteria for recommending TORS resection of post-irradiation OPSCC resectable recurrences at the BoT. Patients who did not meet these criteria were offered other options (transmandibular resection, chemotherapy, immunotherapy. In the period under study, post-irradiation OP-SCC recurrences were not offered re-irradiation as a salvage option in Policlinico Gemelli).

Selection Criteria According to White et al. (2013) [30]—Whole Oropharynx	Proposed Modified Criteria—Base of Tongue Only
No bone involvement	No bone involvement
Anterior extension not considered	No involvement of hard palate, limited (2 cm) extension beyond the lingual V
Tumor spread to be considered not stated (cTNM, rTNM, both?)	Surgery planned on the recurrence (rTNM)
No significant trismus	No significant trismus
Adequate mouth opening	Adequate mouth opening
Base of tongue tumors that cross the midline	At least one lingual artery has to be spareable according to preoperative imaging
Difficult visualization and/or palpation of margins	Preoperative definition through morphological and functional imaging and, in some cases, with histological mapping even when visualization and/or palpation of margins are difficult

**Table 2:** Summary of the clinical history of the 4 study patients who underwent salvage TORS.

Case	Age at Diagnosis (Years)	cT	cN	HPV Status (E6/E7 mRNA on Fresh Sample)	Time to TORS Salvage (Months)	Pre-Op Composite MDADI	rT #	rN #	G, PNI	Post-Op Composite MDADI	Post- TORS Relapse	Dead/Alive	Last Follow-Up After TORS (Months)
1 *	49	4	2c	Positive	65	69	1	0	II, -	73	No	Alive	105
2 *	60	4	2c	Negative	24	68	2	0	III, -	66	No	DOC	72
3	69	1	2b	Negative	18	66	2	0	III, +	-	Yes (time to relapse 3 months)	DOD	11
4	74	3	2c	Negative	9	68	2	2c	II, -	70	No	Alive	96

cT, clinical T classification; cN, clinical N classification; HPV, human papilloma virus; TORS, transoral robotic surgery; MDADI, MD Anderson Dysphagia Inventory; rT, T classification of the recurrence; rN, N classification of recurrence; DOC, dead of other causes; DOD, dead of disease; PNI, perineural invasion, + present, - absent. * Details of Cases 1 and 2 are shown in Figure 1. # The r staging always corresponded to the final pathological (p) staging.

## Data Availability

The data is available when it is requested for motivated reasons.
